# Antimicrobial activity of iron oxide nanoparticle upon modulation of nanoparticle-bacteria interface

**DOI:** 10.1038/srep14813

**Published:** 2015-10-06

**Authors:** Manoranjan Arakha, Sweta Pal, Devyani Samantarrai, Tapan K. Panigrahi, Bairagi C. Mallick, Krishna Pramanik, Bibekanand Mallick, Suman Jha

**Affiliations:** 1 Department of Life Science, National Institute of Technology Rourkela, Odisha 769008, India; 2 Department of Biotechnology and Medical Engineering, National Institute of Technology Rourkela, Odisha 769008, India; 3 Department of Chemistry, Ravenshaw University, Cuttack, Odisha 753003, India; 4 Department of Biotechnology, North Orissa University, Baripada, Odisha 757003, India

## Abstract

Investigating the interaction patterns at nano-bio interface is a key challenge for safe use of nanoparticles (NPs) to any biological system. The study intends to explore the role of interaction pattern at the iron oxide nanoparticle (IONP)-bacteria interface affecting antimicrobial propensity of IONP. To this end, IONP with magnetite like atomic arrangement and negative surface potential (n-IONP) was synthesized by co-precipitation method. Positively charged chitosan molecule coating was used to reverse the surface potential of n-IONP, i.e. positive surface potential IONP (p-IONP). The comparative data from fourier transform infrared spectroscope, XRD, and zeta potential analyzer indicated the successful coating of IONP surface with chitosan molecule. Additionally, the nanocrystals obtained were found to have spherical size with 10–20 nm diameter. The BacLight fluorescence assay, bacterial growth kinetic and colony forming unit studies indicated that n-IONP (<50 μM) has insignificant antimicrobial activity against *Bacillus subtilis* and *Escherichia coli*. However, coating with chitosan molecule resulted significant increase in antimicrobial propensity of IONP. Additionally, the assay to study reactive oxygen species (ROS) indicated relatively higher ROS production upon p-IONP treatment of the bacteria. The data, altogether, indicated that the chitosan coating of IONP result in interface that enhances ROS production, hence the antimicrobial activity.

Increasing bacterial resistance towards traditional/conventional antibiotics is a major global health concern in current era^1^,^2^,^3^,^4^. Some bacterial strains have the potential to produce slime, which facilitates the adhesion and formation of biofilms on any artificial surfaces or implantable devices. Additionally, the formation of biofilms enhances the bacterial resistance by preventing antibiotic action^1^,^2^. Different non-specific interactions like electrostatic, dipole-dipole, H-bond, hydrophobic, and van der Waals interactions are responsible for adhesion of bacteria on any material surfaces creating bacterial biofilms^1^. Hence, before screening any nanoparticle mediated approach as possible antibiotics, the material must have anti-microbial property to reduce the microbial adhesion. In order to achieve the objective, research groups worldwide are focusing on NPs with antimicrobial properties as a promising tool towards controlling microbial adhesion, since the NPs with photocatalytic properties have the potential to reduce biofilm formation^1^.

Nanoparticles, when suspended in biological culture medium, encounter with various biological interfaces resulting from the presence of cellular moieties like DNA, proteins, lipids, flavonoids, polysaccharides etc. Fate of the suspended NP depends upon different physico-chemical properties of nanoparticle and interactions present at the nano-bio interface^5^. Understanding the interactions at nano-bio interface help in adopting approaches for safe use of NPs in biomedical, clinical, and pharmaceutical industries. On other hand, the understanding can help in moderating surface cytotoxic and/or antimicrobial propensities of nanoparticles. Inside biological medium, the interactions between accessible functional groups of NP and biomolecules like lipopolysaccharide (LPS), phospholipid, protein, and lipoteichoic acid (LTA) present over the bacterial envelop or eukaryotic membrane contribute in the interaction pattern at the interface. The same functional groups of biomolecules enhance adhesion of bacteria to different surfaces, and help their proliferation^6^. Hence, the accessible functional groups present at bacterial envelop and NP surfaces, along with the physico-chemical property of the NP, determine the fate of bacteria as well as the NP (whether NP will be compatible or toxic to bacteria). Although various metal and metal oxide NPs like silver, ZnO, Al_2_O_3_, TiO_2_ have been screened as biocidal or antibacterial agents^7^,^8^, consumer products containing some of these NPs possess potential environmental and health risks^1^. It has been reported that among different types of NPs, magnetic NPs due to their biocompatibility, chemical stability, and magnetic behaviour are widely used in biomedical sciences^9^. Magnetic NPs are being used for delivery of drugs to targeted tissues by the application of external magnetic field, which in turn increases the stability of drugs against enzymatic or metabolic degradation^9^,^10^,^11^,^12^,^13^. Due to outstanding magnetic, physico-chemical, thermal, and mechanical properties, magnetic NPs can also be used in other fields, like analytical chemistry^12^, pathogen detection^12^, antigen diagnosis^14^, tissue repair^11^, and hyperthermia^15^. Most of transition metal ions like iron, cobalt, nickel, and their compounds belong to magnetic NPs^16^. Iron oxides coming under magnetic NPs are magnetite (Fe_3_O_4_), maghemite (γ-Fe_2_O_3_), hematite (α-Fe_2_O_3_), and goethite (FeO(OH))^16^.

Nanoparticle, being very small in size, possesses high surface area to volume ratio resulting into surfaces with very high free energy content. To reduce the energy content, surface interacts with possible interactomes present in cell, and become relatively stable entity. However, certain interactions have the energy to overcome band gap barrier of the NP, especially in photocatalytic NP, resulting into generation of electron-hole pair. The free electron, in biological system, results in production of free reactive oxygen species^8^. Additionally, intact IONPs upon contact with air loses its magnetism and monodispersibility^9^. To avoid such problems, various research groups have taken the help of different chemical and biological agents to modify the surfaces, and stop the lose. Surface modification by natural or synthetic polymers create more stable, hydrophilic nanostructures, and provide relatively higher number of variable functional groups on the surface which help in proper binding of interactomes to the nanostructures^17^,^18^. In our work, chitosan was chosen to coat IONP surface, since cationic hydrophilic chitosan polymer is well studied biocompatible molecule with inert chemical nature against air, and possesses amino group which interacts with hydroxide functional group present at the IONP surface^18^.

Thus, the study aims to evaluate the antimicrobial propensity of chitosan coated IONP, which show insignificant anti-microbial activity when not coated with chitosan. To this end, n-IONP was synthesised and coated with positively charged chitosan biomolecules, and their antimicrobial activity against a Gram positive (*Bacillus subtilis*) and a Gram negative (*Escherichia coli*) bacteria were studied using different techniques like bacterial growth kinetics, colony forming unit measurement, ROS, and BacLight fluorescence assay. The data clearly indicate that the interaction pattern at the interface plays a crucial role in determining the antimicrobial activity of IONP. Although, few studies have been done to evaluate the antibacterial activity of IONP against some selective bacteria. However, IONP antimicrobial activity upon coating with biocompatible chitosan molecule and the underlying mechanism of antimicrobial activity have not been explored in detail.

## Results

### Characterization of synthesized nanoparticles

#### X-ray diffraction patterns of the nanoparticles

**Figure 1:**
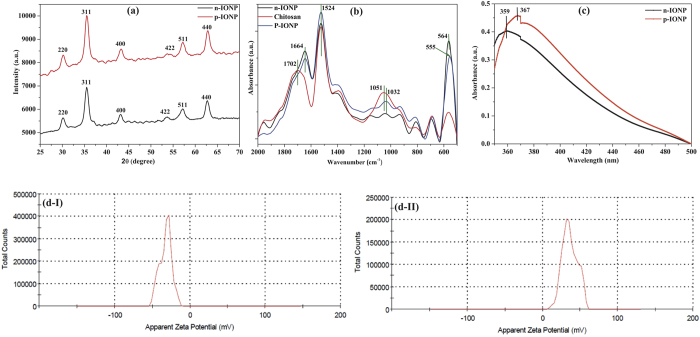
Characterization of n-IONP and p-IONP. (**a**) XRD spectra (**b**) ATR-FTIR absorption spectra, and (**c**) UV-Vis absorption spectra of n-IONP, and p-IONP, (**d**) Zeta potential analysis of n-IONP (Fig. d–I), and p-IONP (Fig. d–II).

Numerous methods have been formulated for synthesis of IONPs, still synthesis of NPs having small and uniform size distribution with good stability to avoid agglomeration is a matter of intensive research in current era. In this study, we have synthesized IONP by chemical precipitation method followed by surface modification with chitosan, a derivative of chitin. The type of synthesized IONP was studied using X-ray diffraction spectroscopy. Figure 1(a) shows the XRD patterns for both n-IONP and p-IONP. The major diffraction peak at 35° (311) in addition to minor peaks at 30° (220), 43° (400), 53° (422), 57° (511), and 62° (440) confirm the spinel structure of iron oxide (magnetite-Fe_3_O_4_ and maghemite- γ-Fe_2_O_3_)^1^, revealing that the synthesized IONP does not contain any other forms of iron oxide such as hematite (α-Fe_2_O_3_), goethite (FeO(OH)), or any iron hydroxides in detectable range^1^. Shan Z. *et al.* has suggested that the lattice parameter (α) for magnetite and maghemite are 8.3960 Å and 8.3515 Å, respectively^19^. In our case, the lattice parameter for n-IONP is 8.3840 Å, which is very close to lattice parameter of magnetite, revealing that the synthesized n-IONP contains predominantly magnetite (Fe_3_O_4_) population. For further confirmation, we have considered the 2θ value of the (311) peak. As reported in various literatures, the standard values of this peak (311) for magnetite and maghemite are at 35.423° and 35.631°, respectively^1^,^19^. Since the diffraction angle for the synthesised NP is 35.47°, more close to magnetite index than maghemite index. It indicates presence of predominantly magnetite (Fe_3_O_4_) lattice with traces of maghemite lattice. From the observations, it is concluded that the synthesized n-IONP is nano-crystal of magnetite (Fe_3_O_4_). The surface modification by chitosan does not affect the crystal structure of Fe_3_O_4_ NP, since the major peaks at 2θ, 30° (220), 35° (311), 43° (400), 57° (511), and 62° (440), correspond to 8.3840 Å lattice, only. The 2θ value for the peak (311) is 35.46, which is basic characteristic feature of Fe_3_O_4_ NP, as described above^1^,^19^. Additionally, the analysis of n-IONP and p-IONP XRD patterns using X’ pert high score software with search and match option reveals that both types of synthesized NPs have Fe_3_O_4_ crystals (JCPDS reference code–75-0033). The particle size of n-IONP and p-IONP was determined using Scherrer equation


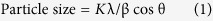


Where λ is the wavelength of X-ray (1.540*10^−10^ m), K = 0.9, proportionality coefficient (shape factor), θ is the Braggs angle and β is the full width at half maximum in radians. On applying this equation, average particle size of n-IONP and p-IONP was calculated to be 10.36 +/− 5.1 nm and 11.37 +/− 5.6 nm, respectively. The size obtained is an average of data obtained from peaks present for n-IONP and p-IONP diffractions, respectively. The XRD spectra obtained in our study are compatible with XRD spectra of different literatures^9^,^20^,^21^,^22^.

#### Bond level characterization of the nanoparticles

Figure 1(b) shows FTIR spectra of n-IONP and p-IONP suspended in deionised water. The data were collected in attenuated mode FTIR using diamond crystal. The presence of Fe_3_O_4_ nano-crystals was confirmed from the peaks at 564 cm^−1^ and 555 cm^−1^, which are due to metal oxygen bond vibrations present in n-IONP and p-IONP nano-crystals, respectively^23^. It is very interesting to observe that there is a peak shift from 564 cm^−1^ to 555 cm^−1^ upon chitosan coating of n-IONP, demonstrating that p-IONP needs lower frequency to vibrate Fe-O bond than n-IONP. Following *Hook’s Law*, the lower frequency of vibration indicates that Fe-O bonds present in p-IONP are less stable than the bond present in n-IONP^8^, hence more prone to produce electron-hole pair. The absorption peak at 1664 cm^−1^ (for n-IONP and p-IONP), 1702 cm^−1^ (for chitosan), and 1524 cm^−1^ (for all) are due to C = O and N-H vibrations, respectively. In the chitosan spectra, the absorption peak at 1051 cm^−1^ is due to vibration of C-O-C bonds^24^, which shifted to 1032 cm^−1^ for chitosan coated IONP (p-IONP). However, shifting of peak from 1051 cm^−1^ to 1032 cm^−1^ with lesser intensity peak at 1032 cm^−1^ is attributed to decreased population of the bond on washing of residual chitosan molecules from bulk. The increased intensities of N-H vibrations for p-IONP confirmed the surface coating of chitosan over negatively charged IONP, indicating a strong interaction between the amino group on chitosan molecules and Fe_3_O_4_ nanoparticle. Thus, the data supports both XRD and UV-Visible data for successful coating of IONPs with chitosan molecule.

#### Surface characterization of the nanoparticles

UV-Visible spectra of both n-IONP and p-IONP are shown in Fig. 1(c). Due to surface plasmon resonance, n-IONP shows characteristic absorption peak at 359 nm, whereas p-IONP shows peak at 367 nm which is in accordance with the reported result by ur Rahman O. *et al.*^25^. The red shift in surface plasmon resonance by 8 nm for p-IONP confirmed chitosan coating on n-IONP. In addition, the shift indicates decrease in energy band gap of NP, i.e. photocatalytic propensity enhanced, strengthening the conclusion of relatively weak Fe-O bond in chitosan coated IONP as drawn from FTIR spectra. The band gap energy for both IONPs is determined using the equation E_bg_ = 1240/λ^26^, where E_bg_ stands for band gap energy in eV, and λ is the wavelength in nanometre. Using this equation, the band gap energy for both n-IONP and p-IONP are found to be 3.4 and 3.3 eV, respectively.

The zeta potential measurements for n-IONP (Fig. 1d-I) and p-IONP (Fig. 1d-II) suspension in deionised water show surface potential of −32.2 mV and +36.3 mV, respectively. p-IONP possesses relatively higher zeta potential magnitude than n-IONP, inferring that chitosan coated IONP is relatively more stable. In addition, reversal of surface potential further support the XRD analysis and surface plasmon resonance spectra data for chitosan coating of IONP. Chitosan is already known for very high adhesive tendency for negatively charged surfaces^27^.

#### Morphological characterization of the nanoparticles

**Figure 2:**
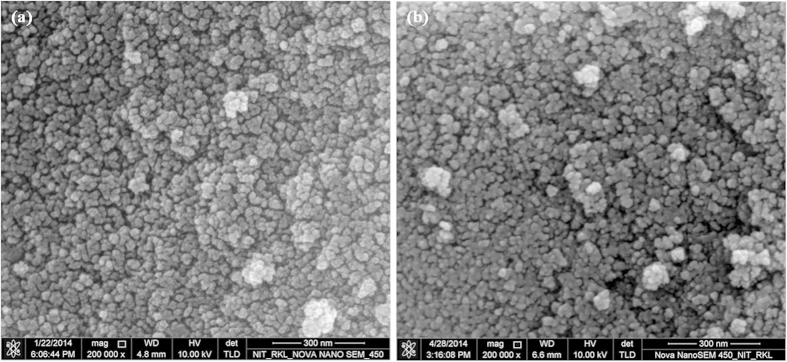
FE-SEM image of n-IONP (Fig. 2a) and p-IONP (Fig. 2b).

The images obtained using FE-SEM, Fig. 2, indicate that both types of IONPs are nearly spherical in shape with size ranging from 8–20 nm, which is very close to the estimated particle size by XRD analysis, i.e. ~11 +/− 5 nm. Both FE-SEM analysis and XRD analysis suggest that the size of the NPs does not change significantly upon chitosan coating. Yu S. *et al.* have reported a similar observation, 8 nm IONP retained its size on coating with poly-(methacrylic acid)^28^. Additionally, slight difference in particle size for n-IONP and p-IONP may be dedicated to instrumental artefact or sampling artefact or both.

#### Effect of the interfaces upon bacterial cell viability

**Figure 3:**
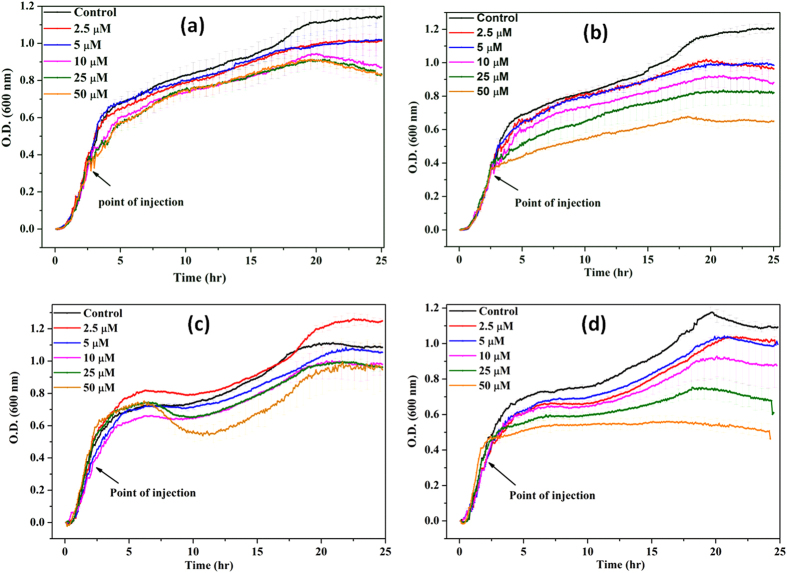
Growth kinetics of *B. subtilis* (Figs. 3a and 3b) and *E. coli* (Figs. 3c and 3d) in absence and presence of different concentrations of n-IONP (Fig. 3a for *B. subtilis* and 3c for *E. coli*) & p-IONP (Fig. 3b for *B. subtilis* and 3d for *E. coli*). Different concentrations of the NPs taken were 2.5, 5, 10, 25, and 50 μM, and injected at the log phase of growth kinetics (shown by arrow). Triplicate experiments were done for each reaction, and the error bar represents the standard error of mean.

**Figure 4:**
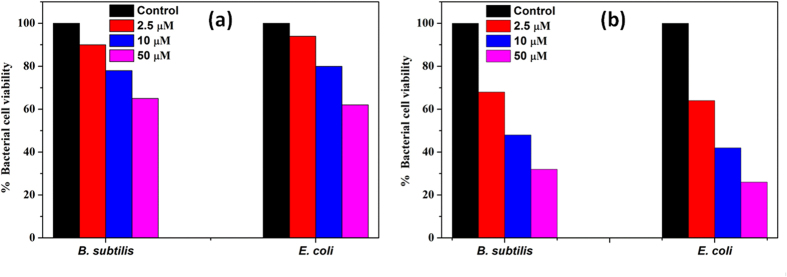
Quantification of bacterial cell viability at different concentrations of n-IONP (Fig. 4a) and p-IONP (Fig. 4b). Colony forming units (CFU) were quantified for both *B. subtilis* and *E. coli* cells, and represented as percentage of viable cells in comparison to colony obtained from untreated culture.

The growth kinetic studies of *B. subtilis* and *E. coli* in presence of different concentrations of n-IONP and p-IONP are shown in Fig. 3. Figure 3a,c display the growth curve of *B. subtilis* and *E. coli*, respectively, in presence of different concentrations of n-IONP. As shown in the figures, insignificant growth inhibition compared to control were observed for the studied concentrations of n-IONP, whereas the inhibition is relatively very dominant for *B. subtilis* (Fig. 3b) and *E. coli* (Fig. 3d) in presence of relative p-IONP concentrations. However, CFU measurements indicate the antimicrobial activity of n-IONP at higher concentrations (Fig. 4). The viability of both bacterial cells reduced by approximately 30% in presence of 50 μM of n-IONP. However, the coated IONP has significant effect on bacterial viability, viability reduced by 70% in presence of 50 μM p-IONP. The data indicates strong antimicrobial propensity of p-IONP against studied bacterial strains. Additionally, the data support the growth kinetic studies observed in presence of n-IONP and p-IONP.

**Figure 5:**
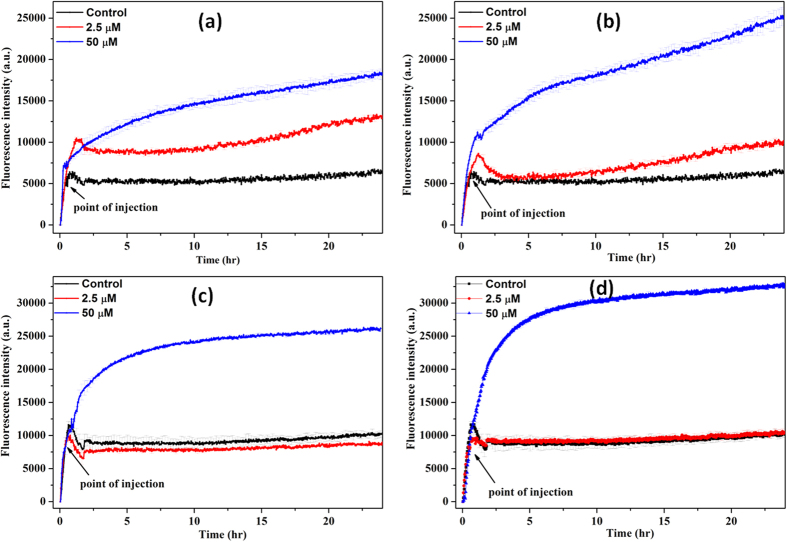
n-IONP and p-IONP induced ROS production. Figure 5(**a,c**) represent change in fluorescence intensity with DCFH-DA oxidation in presence of n-IONP for *B. subtilis* and *E. coli*, respectively. Whereas figure 5(**b**,**d**) represent DCFH-DA oxidation kinetics in presence of p-IONP for *B. subtilis* and *E. coli*, respectively. Each curve represents the average of three independent measurements with corresponding standard error of mean.

Figure 5 shows kinetics of DCFH-DA oxidation on bacterial cell treatment with the NPs. The NPs were added in log phase of bacterial growth. ROS is also produced in culture media in the absence of NP treatment, inferring the production of ROS is natural. Bacteria produces ROS in non-stress condition. The produced ROS in non-stress conditions is counteracted by ROS scavenging enzymes present in bacteria like superoxide dismutase in *E. coli*. However, presence of both n-IONP (Fig. 5a,c) and p-IONP (Fig. 5b,d) resulted in significant increase in the fluorescence intensity, with relatively higher change in p-IONP presence than n-IONP. The change in the fluorescence intensity is directly correlated with the higher amount of ROS production for both *B. subtilis* (Fig. 5a,b) and *E. coli* (Fig. 5c,d) cells. The ROS observation and the growth kinetics study, together, indicated that the ROS production is a reason for antimicrobial activity by both the IONPs. Additionally, it is observed that the amount of ROS produced (as measured from the fluorescence intensity) is higher for p-IONP than n-IONP. The observation rationalized that p-IONP has higher antimicrobial activity than n-IONP.

**Figure 6:**
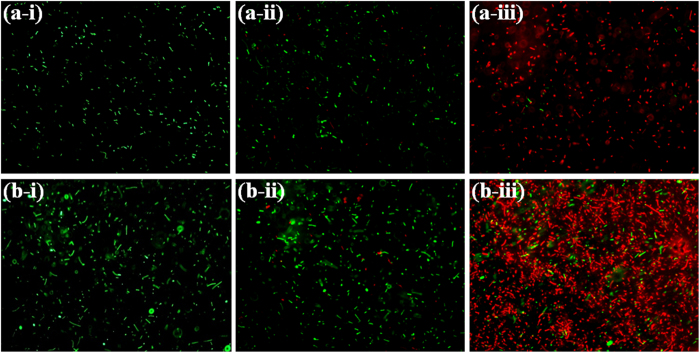
Fluorescence microscopic images of *B. subtilis* and *E. coli* in absence and presence of n-IONP and p-IONP. Intact *B. subtilis* (**a**-**i**)*, B. subtilis* in presence of 50 μM of n-IONP (**a**-**ii**), and *B. subtilis* in presence of 50 μM of p-IONP (a-iii), intact *E. coli* (**b-i**), *E. coli* in presence of 50 μM of n-IONP (**b**-**ii**), and *E. coli* in presence of 50 μM of p-IONP (**b**-**iii**). The scale bars represent for 20 μm.

The antibacterial activity of both NPs resulting from the interaction pattern is further explored using LIVE/DEAD BacLight fluorescence Kit. In principle, LIVE/DEAD BacLight fluorescence kit gives green fluorescence in presence of viable cells, since Syto9, one of the components of the kit, stains the intact membrane of viable cells which has emission in green region. Another component of the kit is propidium iodide which stains dead cells having deformed membrane, and the emission wavelength fall in red region of the visible spectrum^29^. As shown in Fig. 6, untreated (control) bacterial cells showed green fluorescence inferring presence of 100% viable cells. The n-IONP (50 μM) treated samples showed insignificant fraction (~10%) of non-viable bacterial cells, indicating insignificant antimicrobial activity of n-IONP against *B. subtilis* and *E. coli*, at studied concentration. On other hand, p-IONP (50 μM) treated bacterial culture showed 90% of non-viable bacterial cells, which confirmed the significant change in antimicrobial activity of IONP upon chitosan coating.

## Discussion

Studies have been done to demonstrate the antimicrobial activity of IONP^30^,^31^,^32^, still the mechanism behind antimicrobial activity is a matter of intensive research, in present. Chatterjee *et al.*^32^ has demonstrated that IONP has antimicrobial activity against *E. coli*, and the activity increases with increase in concentration of IONP. Borcherding *et al.*^33^, on the other hand, has shown that IONP have no antimicrobial activity. Here, we have extended the studies taking the help of different antimicrobial and biophysical studies to draw a concluding remark against these contrasting statements, and to explore the mechanism behind this antimicrobial activity of IONPs.

Initially, we have synthesized IONP with negative surface potential (n-IONP) having small size and good stability. The surface of the NP was further modified with chitosan to modulate the surface potential and functional groups. Positively charged chitosan molecules are strongly bonded to magnetic n-IONP via electrostatic and/or H-bonding as predominant interactive forces^27^. The chitosan coated IONP has positive surface potential majorly due to the free hydroxyl group (–OH) of chitosan, which interact with the aqueous solution through hydrogen bonds resulting into a stable well dispersed colloidal suspension. Additionally, the coulomb repulsion between the IONPs having positive/negative surface potential also play a role in well dispersivity of the particle^24^.

To explore the effect of interaction pattern at the interface on antimicrobial activity of IONPs, we have carried out growth kinetic, LIVE/DEAD BacLight Bacterial Viability assay, and CFU measurement studies taking both negative and positive surface potential IONPs. From the studies, we found that p-IONP has higher antimicrobial activity than n-IONP. Due to presence of negative potentials on both n-IONP and bacterial surfaces, we hypothesize that the interaction between n-IONP and bacteria would not be strong due to dominant electrostatic repulsion at the interface which is the primary cause of non-attachment of the NP on bacterial cell. However at higher concentration, >50 μM, n-IONP have antimicrobial activity to some extent, as suggested by growth kinetic, LIVE/DEAD BacLight assay, and CFU measurement studies. The finding can be rationalized to the molecular crowding upon increase in the NP concentration, which result into net interactive interaction between nano-bacteria interface. Above a certain concentration of NP, despite the negative surface potential, the particle will be preferentially excluded along with the larger particles or interfaces of same or opposite potentials, like bacterial membrane here. The exclusion, hence, result into the net interactive interactions. The net interactive interaction enhances relative ROS production at the interface, as shown in Fig. 5. Hence, higher concentrations of n-IONP in the culture media is capable of enhancing ROS production, a principal reason for the antimicrobial propensity of metal oxide nanoparticles. The findings support the work of Borcherding *et al.*^33^ provided the concentration of n-IONP is above the critical concentration.

Considering the role of potentials in interaction at the interface, IONP with positive surface potential will have better surface for bacterial attachment with stronger interactions at interface than n-IONP. The stronger interactions will result in relatively enhanced ROS production. Hence, to strengthen the hypothesis, the surface of n-IONP was reversed by coating with positively charged chitosan molecule. Chitosan of different molecular weights, above 25% (w/v), shows significant antimicrobial propensity against both Gram positive and Gram negative bacteria, as reported by Zheng L.-Y. *et al.*^34^. In order to nullify the chitosan mediated antibacterial activity, only 0.02% (w/v) chitosan concentration was used for surface modification of n-IONP. Chitosan at 0.02% (w/v) does not show any antimicrobial activity against studied bacteria (data not shown here). Like predicted, p-IONP inhibit the bacterial cell growth relatively at very low concentration than n-IONP, suggesting that p-IONP have higher antimicrobial propensity. Comparison of the fluorescence intensities in Fig. 5 indicates that the interaction at nano-bacteria interface is relatively stronger for p-IONP than n-IONP. Stronger the interaction, higher is the change in free energy content, resulting into more ROS production. In case of IONP, ROS production follow the Fenton reaction as mentioned below in equations 2 and 3. From the metabolic activity, hydrogen peroxide (H_2_O_2_), which is a toxic oxidant causing DNA and protein damage, is produced in cultures of all aerobic organisms^35^,^36^. *E. coli* produces H_2_O_2_ at the rate 10–15 μM/s during the growth in oxygen rich glucose medium^36^, as also observed in untreated bacterial culture, Fig. 5. However, the produced H_2_O_2_ is counterbalanced by various scavenging enzymes present in cells like superoxide dismutase for *E. coli*. In our study, we measured ROS production in culture by the fluorescent dye, DCFH-DA (described in result section). Upon IONPs dispersion inside the culture media, different oxido-reduction reactions are followed involving both the species present in magnetite, Fe^3+^ and Fe^2+^, resulting into generation of different and more potent reactive oxygen species^36^,^37^. The reactions are known as Fenton reaction or Haber-Weiss cycle.


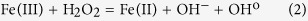



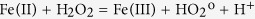


**Figure 7:**
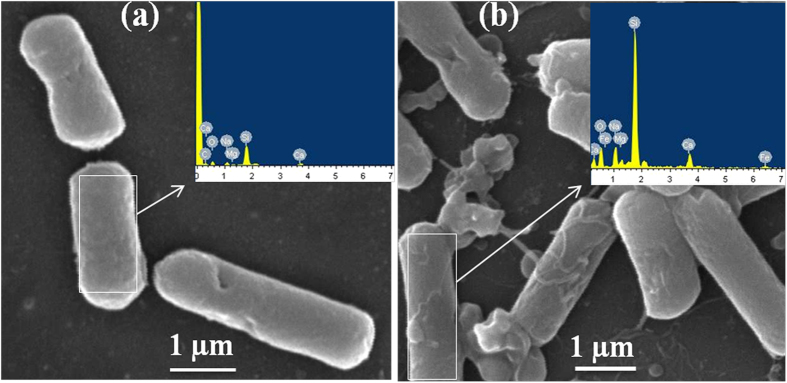
SEM micrographs showing membrane deformation/damage of *B. subtilis* upon p-IONP treatment. (**a**) SEM image of control (without p-IONP treatment), and figure inset shows the EDX spectra of *B. subtilis* surface. (**b**) SEM image of *B. subtilis* cells upon p-IONP treatment, and figure inset shows the EDX spectra of *B. subtilis* surface after p-IONP treatment.

**Figure 8:**
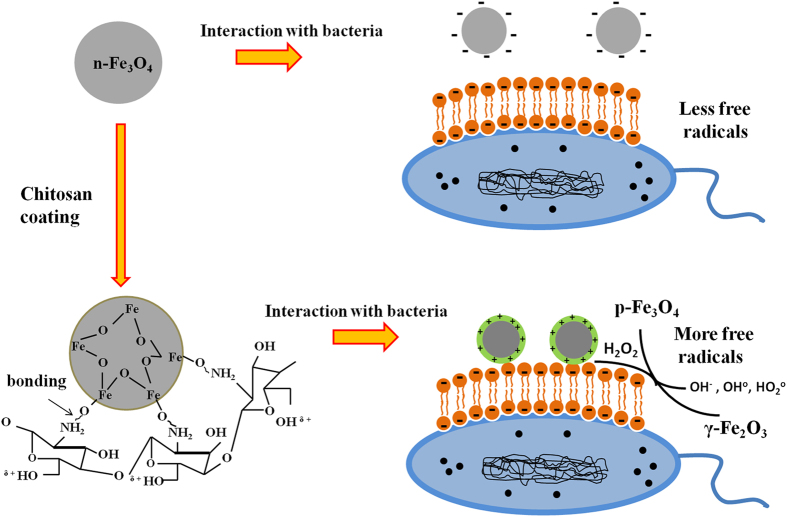
Proposed schematic model elucidating the detail mechanism of IONPs against bacterial cells.

OH° and HO_2_° formed in the process are free radicals. Iron in magnetite (Fe_3_O_4_) NP through a series of reactions is fully oxidized to maghemite (γ-Fe_2_O_3_) causing oxidative stress to bacterial cells, hence bacterial cell death. In contrast, fully oxidized maghemite is relatively stable in culture medium without any further possibility of electronic or ionic transition. Hence, maghemite formed as end product possesses insignificant *in vitro* cytotoxic propensity^37^. Nevertheless, the amount of free radicals formed in the oxido-reduction process are sufficient to put stress on the viable bacterial cells, causing non-viable cells. The amount of ROS produced at the nano-bacteria interface depolarizes the bacterial membranes, causing membrane damage as suggested by BacLight assay (Fig. 6) and in our work with ZnONP^8^. However, we have also checked the membrane depolarization of *B. subtilis* (Fig. 7) using Scanning Electron Microscopy (detail method for sample preparation is in supplementary information), and the interaction between p-IONP and bacteria was confirmed using Energy-dispersive X-ray spectroscope (EDX). Unlike control (inset of Fig. 7a), the EDX spectra of p-IONP treated bacterial surface shows the traces of Fe, confirming the interaction of p-IONP with bacteria catalyses the membrane depolarization (inset of Fig. 7b). Moreover, the bacterial membrane depolarization upon p-IONP treatment, as suggested by BacLight assay, was further confirmed and illustrated in SEM micrograph (Fig. 7). Figure 8 shows the proposed schematic model elucidating the detail mechanism described here to understand the antimicrobial activity followed by p-IONP. Although n-IONP has less antibacterial activity, the growth kinetic study, LIVE/DEAD BacLight assay, and ROS detection studies indicate that the surface modification n-IONP with chitosan makes it more toxic to bacterial cells due to relatively stronger attractive interaction at the interface. Additionally, the cytotoxicity assay using Alamar blue dye following the procedure adopted by Jha, S. *et al.*^38^ (method is in supplementary information, Fig. S2) demonstrated the cytocompatibilty nature of both the nanoparticles, IONP and chitosan coated IONP. The work along with the recently published work from our group, Arakha, M. *et al.* (Scientific Reports, 2015, 5, 09578) indicate that the interfacial potential is not only the determining factor for the bactericidal effects of nanoparticle. In addition to interfacial potential, the interacting functional group at the interface also contribute in the effect through regulating level os ROS production. Hence, adopting the optimized approach, the antibacterial propensity of IONP interface can be modulated using chitosan coating without changing the cytocompatible nature of the nanoparticle.

## Conclusion

The findings conclude that n-IONP has antimicrobial activity at relatively very high concentrations. The activity can be further moderated by changing the surface potential and accessible surface functional groups. The changes cause change in interaction pattern at the nano-bio interface, hence play crucial role in determining the antimicrobial propensity of IONPs. However, the enhanced production of ROS because of the interaction potential at the interface is the principal cause for antimicrobial propensity of the NPs. As a conclusion, the interaction pattern at the nano-bio interface plays vital role in determining the antimicrobial activity of metal oxide nanoparticles.

## Methods

Ferrous chloride tetrahydrate (FeCl_2_.4H_2_O) and ferric chloride hexahydrate (FeCl_3_.6H_2_O) were purchased from Sigma Aldrich Pvt. Ltd., Germany. Sodium hydroxide (NaOH) was purchased from Merck, India, whereas nutrient broth, nutrient agar, and chitosan were purchased from HIMEDIA, India. 2′, 7′-Dichlorodihydrofluorescein diacetate (DCFH-DA) used for ROS study was purchased from Cayman Chemicals, USA. All the mentioned chemicals were of analytical grade, and used for experimental works without further purification. *Bacillus subtilis* (MTCC-736) and *Escherichia coli* (MTCC-443) strains were purchased from the Institute of Microbial Technology (IMTECH), Chandigarh, India.

### Synthesis of IONPs

The n-IONP was synthesized from ferrous chloride tetrahydrate (FeCl_2_.4H_2_O) and ferric chloride hexahydrate (FeCl_3_.6H_2_O) by co-precipitation method, following the protocol described by Bellova *et al.*^39^ with some modifications. Required amounts of 0.1 M FeCl_2_.4H_2_O and 0.2 M FeCl_3_.6H_2_O were added to 100 mL of deionised water, and stirred using a magnetic stirrer until a homogeneous solution was formed. The solution was sealed and heated at 60 °C for 15–20 min in a water bath followed by the addition of 14 mL of 25% sodium hydroxide (NaOH). The black precipitate formed upon completion of the reaction was centrifuged at 7000 rpm for 15 min, and washed three times with deionised water, followed by drying at 60 °C to get the powder form of n-IONP. For surface modification of n-IONP, we followed the protocol adopted by Samani S. M. *et al.*^40^. In Brief, 20 mg of chitosan was dissolved in 100 mL of deionised water containing 1 M of acetic acid, and vortexed for 5 minutes. 70 mg of synthesized n-IONP was dissolved in the above prepared chitosan solution, and kept overnight (~18 hours) on a magnetic stirrer at room temperature (25 °C). During the process, chitosan molecules absorbed over the n-IONP surface resulting into colour change from black to brown. After the change in colour, suspension was centrifuged at 7000 rpm for 30 minutes, and pellet was collected. The pellet was further washed two times with deionised water to remove unabsorbed chitosan molecules and traces of acetic acid, and dried to powder at 70 °C to obtain p-IONP.

### Characterization of n-IONP and p-IONP

The XRD patterns of both iron oxide and chitosan coated iron oxide NPs were recorded on a X-ray diffractometer (Ultima IV model Rigaku, Tokyo, Japan) using CU-Kα radiation at a voltage of 40 KV and a current of 40 mA with a scan rate of 20°/min and step size of 0.05° over 2θ range of 25° to 70°. The different phases present in the synthesized sample were evaluated using X′-pert high score software having search and match facility. The Field Emission Scanning Electron Microscope (FE-SEM, Nova Nano SEM 450, FEI company, Netherland) was employed to analyze the morphological features of synthesized IONPs at an accelerating voltage of 10 KV upon gold coating for 3 mins. The UV-Vis spectrophotometer (Cary 100, Agilent Technology, Singapore), was employed to study the surface plasmon resonance properties of both n-IONP and p-IONP at desired dilutions using deionised water. The Fourier transform infrared (FTIR) spectra of both types of the IONPs along with chitosan were recorded on a alpha platinum attenuated total reflectance (ATR)-FTIR spectrophotometer (Bruker, Germany) in ATR mode with 128 scans and 8 cm^−1^ resolution in a range of 2000–500 cm^−1^ on diamond crystal, whereas the surface potential of both IONPs was measured using zeta analyzer (Malvern Zetasizer Nano ZS90, Netherland). Moreover, the concentration of Fe present in our synthesized IONP samples were determined using atomic absorption spectrophotometer (Perkin Elmer AA200, Singapore). Specific cathode lamps were employed and the instrument tuned to corresponding wavelengths. Air-acetylene flame was used for atomization of IONP suspension. Initially, the flame absorptions were calibrated by using respective standard solutions in the range of 1–2 mg/L. Following the calibration, concentration of the synthesized IONP samples were found 18.1 ppm of Fe. On applying the empirical formula of magnetite (Fe_3_O_4_) present in the nanoparticles, since our synthesized iron oxide is predominantly magnetite as reported by XRD results, the equivalent concentration of iron oxide in suspension were estimated to be 101 μM. The different concentrations in reactions were obtained by dilution with respective buffers or nutrient broth.

### Effects of IONPs interface on bacterial cell viability

#### Growth kinetic analysis

Initially, effects of interaction pattern at IONP-bacteria interface is studied by following growth kinetics of *B. subtilis* and *E. coli* in absence and presence of different n-IONP and p-IONP concentrations. The mother cultures of test organisms were prepared in nutrient broth taking loop full of bacteria from the specified slant culture, and cultured overnight at 37 °C and 150 rpm agitation. Different concentrations of both n-IONP and p- IONP taken were 2.5, 5, 10, 25, and 50 μM. The IONP stock solution was prepared by dispersing IONP in sterilized nutrient broth and sonicated for 10 minutes followed by UV radiation sterilization before use. The reaction mixtures without NPs were taken as controls. Briefly, 20 μL of bacterial mother cultures were added to the different reaction mixtures prepared in 96-well plate, and the reaction volumes were adjusted by adding nutrient broth to a final volume of 300 μL with nanoparticle. The growth kinetic studies were performed by measuring optical density (O.D.) at 600 nm using plate reader (Synergy H1 hybride reader, Biotek, USA) at regular time interval. At approximately mid log phase of bacterial growth, respective concentrations of NPs were added to the respective reaction mixtures. Upon addition of the NP, data collection for growth curve was immediately started with dead time of 10 min.

#### CFU measurement

The number of viable cells were quantified by measuring colony forming units (CFUs) for both types of bacteria upon treatment with different n-IONP and p-IONP concentrations. For CFU measurement, 10 μL of sample from the stationary phase of growth kinetics was taken from different reaction mixtures having different n-IONP and p-IONP concentrations (2.5, 10, and 50 μM), and spread on the nutrient agar plates after 10000 times dilution in autoclaved distilled water. The plates were incubated overnight at 37 °C. The number of viable cells after treatment with different IONPs were quantified, and compared with positive control (culture without NP) to evaluate the antimicrobial propensity of IONPs.

#### ROS detection

The production of ROS, predominantly responsible for the toxicity of NPs, was evaluated using 2′, 7′-dichlorodihydrofluorescein diacetate (DCFH-DA). DCFH-DA, a peroxynitrite indicator, is having the potential to detect both nitric oxide and hydrogen peroxide (considered as ROS) inside as well as outside of the cells^41^. The bacterial cells were treated with DCFH-DA (200 μM), and fluorescence emission at 523 nm was measured using Synergy H1 hybrid reader (Biotek, USA) with an excitation at 503 nm. Different n-IONP and p-IONP concentrations (0, 2.5, and 50 μM) were added to respective wells at log phase of the growth kinetics. ROS variation was determined comparing the fluorescent intensities of different wells with that of positive control (without NPs treatment). For error bar these experiments were done in triplicate.

#### LIVE/DEAD BacLight fluorescence assay

The fluorescence assay is one of the important method to visualize the effect of interaction pattern on antimicrobial propensity of the NPs in the bulk culture. The fluorescence microscope (Olympus IX71, Germany) with 20X objective lens was employed for imaging bacteria cells (control) and NPs (50 μM) treated bacterial cells to distinguish viable cells from dead cells with the help of LIVE/DEAD BacLight Bacterial Viability Kit (L7007, Molecular probes, invitrogen). In brief, 30 mL of each cultures, *B. subtilis* and *E. coli*, were prepared in separate flasks by inoculating 1 mL of overnight culture in 29 mL of nutrient broth. At mid log phase of bacterial growth, the NP solutions were added to the final concentration of 50 μM NPs, and allowed to grow till late log phase. From these cultures, 25 mL of each bacterial solutions were centrifuged at 7000 rpm for 15 minutes. The supernatant were discarded, and the pellets were suspended in 2 ml of HEPES buffer (10 mM, pH 7.4, containing 150 mM NaCl). 1 mL of the bacterial cell suspensions were added to 20 mL of HEPES buffer in two separate tubes. Both samples were incubated at room temperature for one hour (mixing every 15 minutes). The suspensions were centrifuged at 7000 rpm for 15 minutes, pellets were collected and resuspended in 20 mL of HEPES buffer, and centrifuged at 7000 rpm for 15 minutes. Finally, the pellets were resuspended in 10 mL of HEPES buffer in separate tubes, and optical density was measured at 670 nm. 3 μL of dye mixture (equal volume of component A and component B of the kit) was added to each 1 mL of the prepared bacterial samples, and incubated in dark for 15 minutes after proper mixing of bacterial suspensions. Fluorescence images were taken by trapping 5 μL of stained bacterial samples between a slide and cover slip.

## Additional Information

**How to cite this article**: Arakha, M. *et al.* Antimicrobial activity of iron oxide nanoparticle upon modulation of nanoparticle-bacteria interface. *Sci. Rep.* **5**, 14813; doi: 10.1038/srep14813 (2015).

## Supplementary Material

Supplementary Information
