# Supplementary material for: Antimicrobial activity of iron oxide nanoparticle upon modulation of nanoparticle-bacteria interface
Source: Sci Rep. 2015 Oct 6;5:14813. doi: 10.1038/srep14813 (PMC4594095; doi:10.1038/srep14813)
Supplement: Supplementary Information [file srep14813-s1.doc]

Supplementary Information

**Antimicrobial activity of iron oxide nanoparticle upon modulation of nanoparticle-bacteria interface**

Manoranjan Arakha^a^, Sweta Pal^a^, Devyani Samantarrai^a^, Tapan K. Panigrahi^d^, Bairagi C. Mallick^c^, Krishna Pramanik^b^, Bibekanand Mallick^a^, Suman Jha^a*^

^a^Department of Life Science, National Institute of Technology Rourkela, Odisha 769008, India.

^b^Department of Biotechnology and Medical Engineering, National Institute of Technology Rourkela, Odisha 769008, India.

^c^Department of Chemistry, Ravenshaw University, Cuttack, Odisha 753003, India.

^d^Department of Biotechnology, North Orissa University, Baripada, Odisha 757003, India.

**Methods**

**Characterization of IONPs by Transmission Electron Microscopy**

The morphology of synthesized IONPs was studied using JEM 1400 Transmission Electron Microscopy (TEM) (JEOL, Japan) at an accelerating voltage of 120 KV. The IONP samples were dispersed in deionised water, filtered using 0.22 μM cut-off filter prior to sonication, and mounted on copper grid for imaging using TEM.

**Interaction between IONP and bacteria**

The interaction between the IONPs and bacteria was studied using Scanning Electron Microscopy (Jeol-JSM- 6480 LV SEM, Japan) and Energy-dispersive X-ray spectroscopy (EDX) equipped with the SEM. For both the studies sample preparation was done following the protocol adopted by Arakha *et al* ^1^. In brief, IONP treated bacteria samples were collected from the stationary phase of growth kinetics and centrifuged at 5000 rpm for 5 minutes followed by washing the pellets using 1 X PBS buffer, twice. The pellets were resuspended again in PBS buffer, and one drop of resuspended bacteria sample was put on the slide. The slides were dried by incubating at 37 ^o^C. Upon drying, bacteria cells were fixed by flooding 2.5 % glutaraldehyde (Merck, India) and kept in incubator for overnight at 37 ^o^C. The slides were again flooded with 1% tannic acid (Himedia, India), and left for 5 minutes followed by washing with distilled water and dehydration using increasing concentration of ethanol (30%, 50%, 70%, 90%, and 100%). The slides were coated with platinum before image collection using SEM. Upon SEM scanning, EDX analysis of bacterial surface was done. In EDX, the peaks of Fe in inset of fig. 7b confirm the presence of p-IONP on the surface of bacterial cells. The other peaks in both the EDX spectra (inset of Fig. 7a & 7b) are due to the elemental composition of bacterial membrane, media, buffer, and the slides used for preparation of the samples.

**Cytotoxicity of IONPs**

The cytotoxicity of iron oxide nanoparticles (IONPs) against Human Embryonic Kidney (HEK 293, NCCS Pune, India) cell line was measured using Alamar blue dye reduction assay, following the protocol adopted by Jha S. *et al* ^2^. In brief, 5000 cells/well were seeded in a 96-well plate with DMEM medium (Himedia, India), supplemented with 10 % fetal bovine serum (FBS). After 24 hours of incubation at 37 ^o^C with 5% CO_2_ in an incubator, the culture medium was replaced with fresh media containing the IONPs at different concentrations (0, 2.5, 5, 10, and 25 µM), followed by further incubation of 24 hours. 10% (v/v) Alamar blue dye (Invitrogen, USA) was added to the respective wells and fluorescence intensity generated due to reduction of Alamar blue fluorescent dye by live cell metabolism was measured at emission wavelengths of 590 nm, with excitation at 544 nm. Percentage of viable cells in IONP treated samples with respect to control was calculated from the ratio of fluorescence intensity in treated cells to untreated cells. Triplicate experiments were performed for each reaction, and the error bar represents the standard error of mean.

**
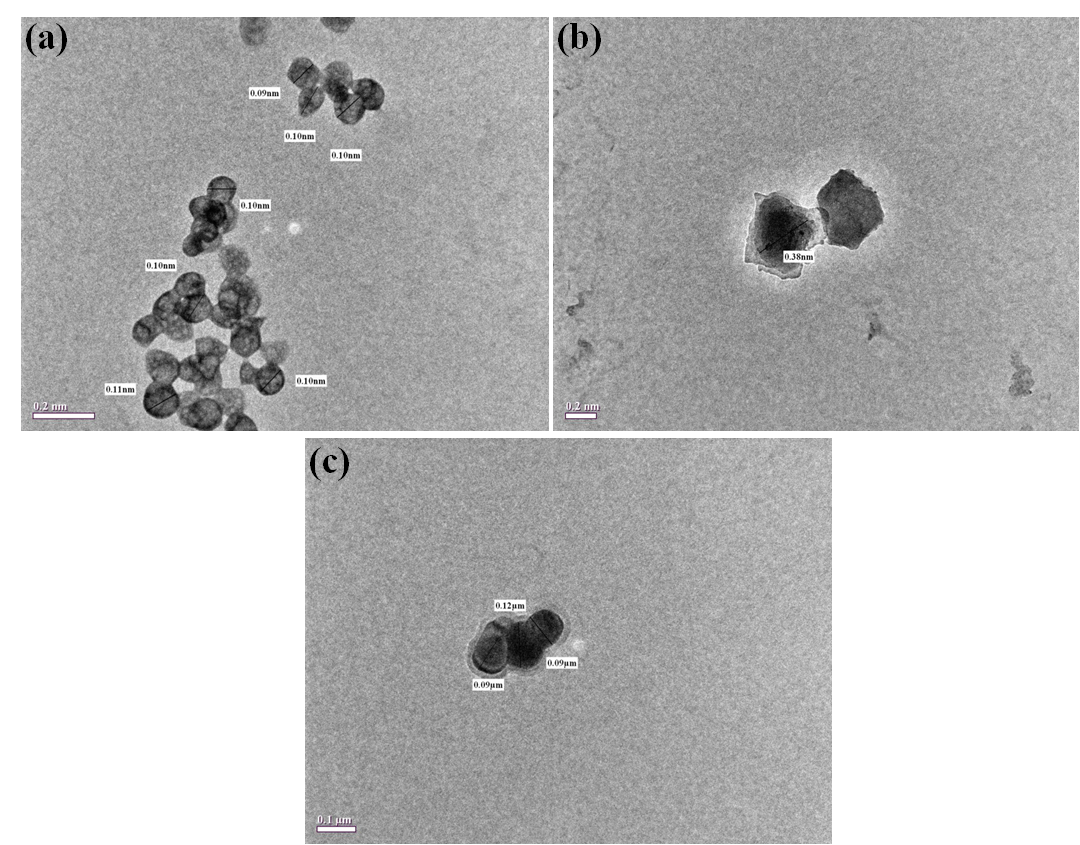
**

Figure S1 ǀ TEM micrograph of n-IONP (a) and p-IONP (b) showing the ultra-fine iron oxide nanoparticles having size of 0.1 nm (a) and 0.3 nm (b), and nanoparticles of size ~ 90 nm IONP (c).


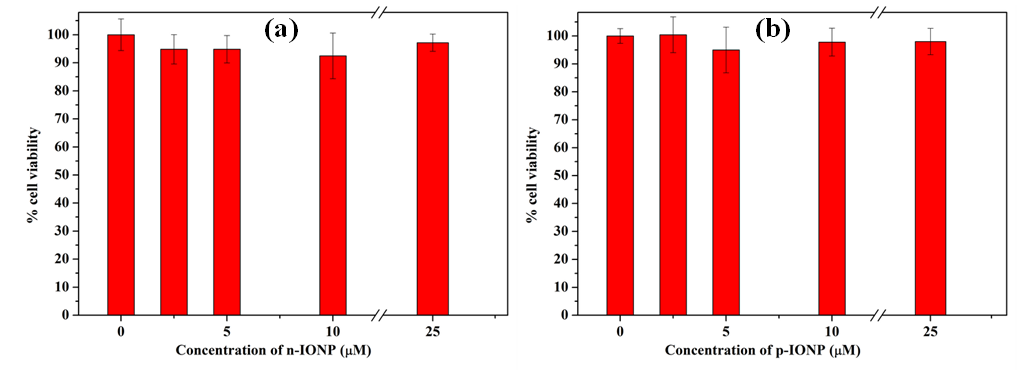


Figure S2 ǀ Cytotoxicity of both n-IONP and p-IONP against Human Embryonic Kidney 293 (HEK 293) cell line using by Alamar blue dye reduction assay. Both the nanoparticles show cytocompatible nature against the studied cell line.

References

1 Arakha, M., Saleem, M., Mallick, B. C. & Jha, S. The effects of interfacial potential on antimicrobial propensity of ZnO nanoparticle. *Sci rep* **5** (2015).

2 Jha, S. *et al.* Mechanism of amylin fibrillization enhancement by heparin. *J. Biol. Chem.* **286**, 22894-22904 (2011).
